# Smoking and incidence of glaucoma: The SUN Cohort

**DOI:** 10.1097/MD.0000000000005761

**Published:** 2017-01-10

**Authors:** Mónica Pérez-de-Arcelus, Estefanía Toledo, Miguel Á. Martínez-González, Nerea Martín-Calvo, Alejandro Fernández-Montero, Javier Moreno-Montañés

**Affiliations:** a Department of Preventive Medicine and Public Health, School of Medicine-Clinic, University of Navarra, Pamplona, Navarra; b CIBER Fisiopatología de la Obesidad y Nutrición (CIBER obn), Instituto de Salud Carlos III (ISCIII), Spanish Government, Madrid; c IdiSNA, Navarra Institute for Health Research; d Department of Ophthalmology, School of Medicine-Clinic, University of Navarra, Pamplona, Navarra, Spain.

**Keywords:** cohort study, glaucoma, smoking, SUN

## Abstract

Smoking is a serious global public health concern that has been related to many chronic diseases. However, the effect of smoking on eye disorders has been less studied. The aim of this cohort study was to assess the association between current tobacco smokers and the risk of developing glaucoma and furthermore to evaluate the relationship between passive or former smokers and glaucoma.

In this prospective and dynamic cohort, 16,797 participants initially who were found not to have glaucoma were followed up for a median of 8.5 years. Validated data on lifestyle, including tobacco consumption, were assessed at baseline. Information about new diagnosis of glaucoma was collected by follow-up questionnaires every 2 years. The outcome was the incidence of self-reported glaucoma during the follow-up. A subsample was used to validate the glaucoma diagnosis.

During the 8.5 years of follow-up, 184 new glaucoma cases were identified. Current smokers had a significantly higher risk of glaucoma compared to participants who had never smoked after controlling for potential confounders (Hazard ratio [HR] 1.88 [95% coefficient interval (CI): 1.26–2.81]; *P* = 0.002). A nonsignificant increased risk was found among former smokers (HR 1.27 [95% CI: 0.88–1.82]; *P* = 0.198). When we assessed the exposure as per the number of cigarette pack-years, a dose–response relationship between pack-years and the risk of glaucoma was found (HR for the 5th quintile versus the 1st quintile: 1.70 [95% IC: 1.10–2.64], *P* for trend, 0.009). However, no relationship was found between passive smokers and glaucoma. (HR 0.67 [95% CI: 0.37–1.21]; *P* = 0.189).

Our results suggest a direct association between current smokers and the incidence of glaucoma. In particular, this association was related to the number of pack-years, which was not found in the case of former smokers nor in the case of passive smokers.

## 1 Introduction

According to the World Health Organization, smoking has become a serious problem for public health. Among the approximately 1.3 billion smokers in the world, over 6 million die annually due to tobacco exposure.^[1]^ Although the effect of smoking on eye disorders has been less studied than the smoking effects on cancer, cardiovascular disease, and other major noncommunicable disease, in some studies, cigarette smoking has been related to several eye diseases such as glaucoma.^[2–4]^

Glaucoma includes a group of disorders characterized by progressive deterioration of the optic nerve associated with loss of the field of vision. Primary open-angle glaucoma (POAG), which affects 60 million people all over the world,^[5]^ is the most prevalent form of that disease.^[5]^ It is the 2nd-leading cause of blindness worldwide and the major cause of irreversible blindness. Currently, the only well established modifiable risk factor is an elevated intraocular pressure (IOP),^[6–8]^ but its pathogenesis is still poorly understood. Many investigators believe that POAG has a vascular origin due to a compromised blood flow to the optic nerve head,^[9]^ and it is known that cigarette smoking contributes to vascular disease by occluding arterial lumina with atherosclerotic plaques and intimal thickening.^[10]^ In addition, trabecular meshwork cells (TMC) and retinal ganglion cells (RGC) damage, involving inflammation and apoptosis mechanisms, has been proven in glaucoma, and it has been demonstrated how smoking can cause high oxidative stress because of oxidizing agents that produce free radicals.^[11]^ Consequently, smoking might be involved in the pathogenesis of POAG, along with other risk factors.

In contrast to the already known risk factors for POAG, such as advanced age, a family history of glaucoma and African ethnicity, tobacco smoking is a modifiable risk factor. This is important because it gives us the possibility to control the disease, at least partially, through modifying habits. This idea would support existing public health efforts toward reinforcing no-smoking campaigns. However, existing data on the association between tobacco smoking and glaucoma are controversial.^[12,13]^ A recent systematic review concluded that heavy smoking may increase the risk of POAG, even though the authors indicated that future studies that will specifically assess the number of pack-years were still needed.^[14]^ Thus, our aim was to evaluate the association between tobacco smoking and the risk of developing glaucoma in the Seguimiento Universidad de Navarra (SUN) cohort.

## 2 Materials and methods

### 2.1 Study population

**Figure 1:**
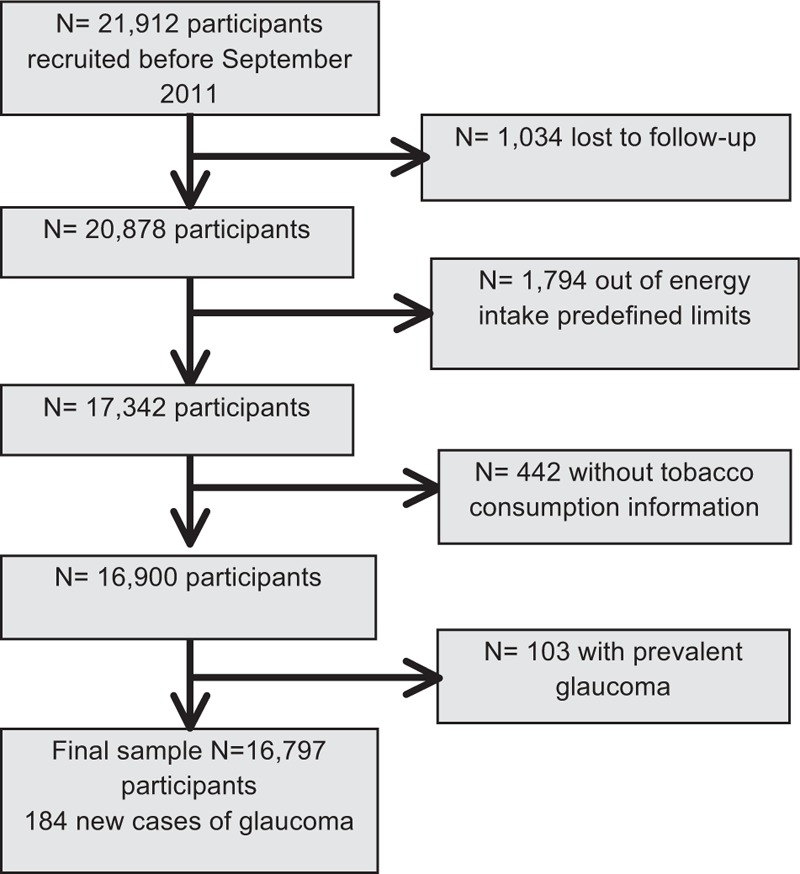
Flow-chart of participants in the Seguimiento Universidad de Navarra (SUN) Project, 1999 to 2010.

The SUN study is a multipurpose dynamic Spanish cohort of university graduates. The study methods have been previously published in detail in other papers.^[15]^ In summary though, information about both exposure and outcome was gathered by mailed questionnaires collected biennially. People who do not reply to follow-up questionnaires are sent up to 5 additional mailings. The recruitment of participants started in December 1999 and it is still on-going. The overall follow-up rate is 89.2%. Before September 2011, 20,878 participants had answered the baseline questionnaire and were included in the sample. A total of 4081 participants were excluded for different reasons: 1742 participants because contact was lost before follow-up, 1794 participants because they had a total energy intake that fell outside of the predefined limits (<800 or >4000 kcal/day for men, and <500 or >3500 kcal/day for women),^[16]^ 442 due to the absence of information about tobacco smoking, and 103 due to prevalent glaucoma. Thus, the final sample size for the analyses included 16,797 participants (Fig. 1).

The study was approved by the Human Research Ethical Committee at the University of Navarra. Voluntary completion of the 1st questionnaire was considered to imply informed consent.

### 2.2 Smoking assessment

Smoking information was assessed in the baseline questionnaire. Information about smoking status was gathered by means of the question: “have you smoked 100 or more cigarettes in your life?” and it was ascertained whether the participants were former, current, or nonsmokers. Those participants who answered positively were asked for the mean number of cigarettes consumed per day at some specific periods of their life. Besides, former smokers were asked how long ago they gave up smoking. The number of pack-years consumed by the smokers in the cohort was estimated using this information. We used the pack-years definition according to the National Cancer Institute Dictionary of Cancer Terms; pack-years were calculated by multiplying the number of packs of cigarettes smoked per day by the number of years the person has smoked. For example, 1 pack year is equal to smoking 1 pack per day for 1 year, or 2 packs per day for half a year, and so on. No other tobacco characteristics such as type, quality, filter, or nicotine content were evaluated.

Passive smokers were defined as people that answer positively to the question: “Have you lived or worked with a smoker in the same room for more than a year?” No other variables were taken into account in this analysis.

### 2.3 Dietary exposure assessment

The dietary exposure was ascertained through a 136-item semiquantitative food-frequency questionnaire previously validated in Spain.^[17]^ Food consumption was calculated as the self-reported frequency of food items multiplied by the nutrient composition of specified portion sizes where frequencies were measured in 9 categories (never or almost never, 1–3 times/month, once/week, 2–4 times/week, 5–6 times/week, once/day, 2–3 time/7day, 4–6 times/day, and 6+ times/day) for each food item. Nutrient intake scores were computed using a bespoke computer program specifically developed for this aim. A trained dietitian updated the nutrient data bank using the latest available information included in food composition tables for Spain.^[18,19]^

### 2.4 Assessment of other covariates

The baseline assessment also included other questions (totaling 46 items for men and 54 items for women). Sociodemographic (eg, gender, age, marital status, and employment status), anthropometric (eg, weight and height), lifestyle and health-related habits (eg, physical activity during leisure time), psychological characteristics (eg, self-perceived personality traits), and medical history (eg, prevalence of chronic diseases and medication use) variables were collected.

The validated physical activity questionnaire included information about 17 activities.^[20]^ To quantify the volume of activity during leisure time, an activity metabolic equivalent (MET) index was computed by assigning a multiple of the resting metabolic rate (MET score) to each activity, and the time spent on each of the activities was multiplied by the MET score specific to each activity, and then all activities were added up to obtain a value for the overall weekly MET-h.^[21]^

Body mass index (BMI), defined as weight (kg) divided by the square of height (m^2^), was computed using self-reported information on weight and height from the baseline questionnaire. Validity of self-reported weight has been assessed in a subsample of the cohort.^[22]^

### 2.5 Outcome assessment

Glaucoma was assessed by means of a question included in the follow-up questionnaires. Participants responded to the question: “Have you ever been diagnosed with glaucoma by a health professional?” The question also specified the date of diagnosis. The validity of a self-reported diagnosis of glaucoma in our cohort is supported in part by the high educational level and strong motivation of this cohort. Moreover, more than 50% of the participants in the cohort are health professionals with a good medical knowledge. However, the validity of self-reported glaucoma diagnosis was also assessed in a subsample of our cohort. The diagnosis of glaucoma was evaluated by an experienced ophthalmologist without having seen the answers to the questionnaires. This validation study showed an adequate validity of the self-reported diagnosis of glaucoma: the Kappa value was 0.85 (95% coefficient interval [CI], 0.834–0.872). The sensitivity was 0.83 and the specificity 0.99.

### 2.6 Statistical analysis

Cox regression models were fit to assess the relationship between smoking status (never/former/current smoker) or cigarette pack-years and the incidence of glaucoma. Hazard ratios (HRs) and their 95% CI were calculated considering the never smoker status as the reference category. Participants contributed to the follow-up period up to the date of return of their last questionnaire, death, or diagnosis of glaucoma, whichever came first.

Potential confounders included as covariates in the multiple Cox models were age, sex, body mass index (kg/m^2^), omega 3: omega 6 ratio (quintiles), hypertension, type 2 diabetes, physical activity (tertiles), coffee consumption (4 categories), alcohol intake (quintiles), and adherence to the Mediterranean diet.

All *P* values presented are 2-tailed; *P* < 0.05 was considered a priori as statistically significant. All analyses were performed with STATA/SE 13.1 software (College Station, TX: StataCorp LP).

## 3 Results

During the follow-up period (median 8.5 years), 184 incident cases of glaucoma were identified from a total number of 144,313 person-years.

Baseline characteristics of the participants according to smoking status are presented in Table 1. The mean age of participants was 39 years (SD: 12). We included in our analyses 7920 nonsmokers, 3729 former smokers, and 5160 current smokers. Current smokers were more likely to report coffee and alcohol consumption, higher adherence to the traditional Mediterranean dietary pattern, and were also more likely to have hypertension and diabetes. Former smokers were older and the proportion of men was higher in this category.

**Table 1:**
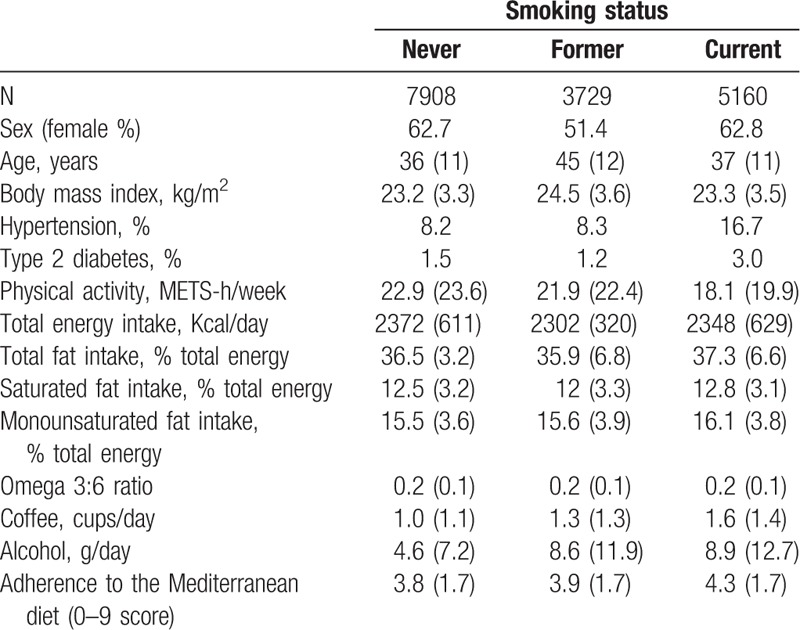
Baseline characteristics of the participants according to smoking status (mean and standard deviation unless otherwise stated), Seguimiento Universidad de Navarra (SUN) Project 1999 to 2011.

Current smokers showed a significant higher risk of developing glaucoma compared to nonsmokers after controlling for potential confounders (HR 1.88 [95% CI: 1.26–2.81]; *P* = 0.002). Besides, former smokers had a nonsignificant higher risk of developing glaucoma compared with never-smokers in the multiple-adjusted model (HR 1.27 [95% CI: 0.88–1.82]; *P* = 0.198) (Table 2).

**Table 2:**
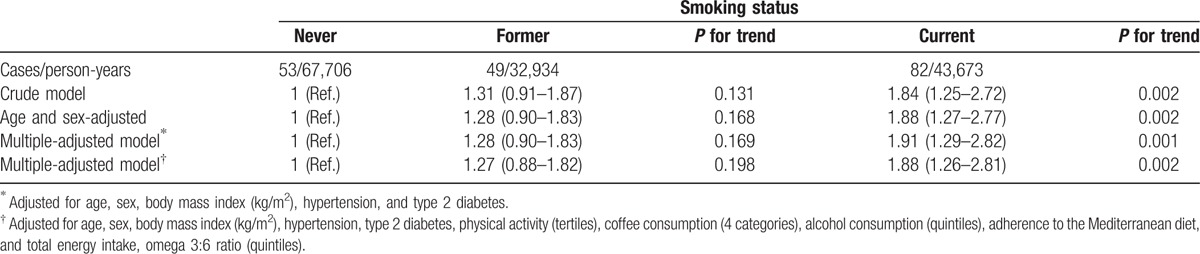
Hazard ratios (95% confidence interval) of glaucoma according to smoking status.

When the exposition was assessed per number of cigarette pack-years consumed, we found a dose–response relationship which supports that the risk of developing glaucoma increased monotonically with the number of pack-years consumed. As presented in Table 3, participants in the 5th quintile of cumulative exposure to smoking (>20.5 pack-years) showed a 70% higher risk of developing glaucoma compared to never-smokers (95% CI: 1.10–2.64; *P* for trend = 0.009).

**Table 3:**
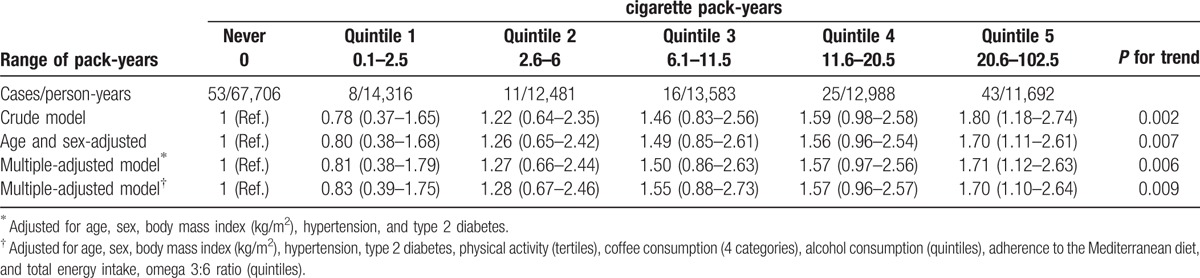
Hazard ratios (95% confidence interval) of glaucoma according to cigarette pack-years consumed.

Passive smokers, noncurrent and nonformer smokers (n = 2897), were compared with nonpassive, noncurrent, and nonformer smokers (n = 4958). The results suggest no statistical significance between either groups with HR = 0.65 (95%CI: 0.35–1.18, *P* = 0.157).

## 4 Discussion

In this large prospective study, we found that current smoking was significantly associated with the risk of developing glaucoma and that this association was even stronger among heavy smokers. To our knowledge, there are only a limited number of studies that have analyzed the association between smoking and the incidence of glaucoma, and furthermore, their conclusions are controversial.^[23–31]^ Most of the results come largely from case–control studies, yielding both positive^[14,23–27]^ and null^[28–31]^ associations. In a cohort of African-American women, Wise et al^[32]^ reported that smoking might be associated with an increased risk of early-onset POAG, but the results did not reach statistical significance (*P* = 0.28). In contrast, a prospective follow-up study of Female Nurses from 1980 to 1996 and Health Professionals from 1986 to 1996 showed that neither current smokers nor former smokers were at greater risk of developing POAG than those who had never smoked, and that heavier smoking did not increase the risk of POAG.^[12]^ It is noteworthy that most studies did not consider the number of pack-years smoked. In a case–control study, POAG was associated with heavy smoking (40 pack-years or more, OR = 3.93, 95% CI: 1.12–13.80, *P* = 0.03) but not with moderate (20–40 pack-years) or light smoking (<20 pack-years).^[33]^ On the contrary, in the Nurses’ Health Study and in the Health Professionals Follow-Up Study, Kang et al^[12]^ found a borderline significant inverse dose effect association only among former smokers. In a recent systematic review, the authors indicated that recent studies suggest that heavy smoking may increase the risk of POAG.^[14]^

Two meta-analyses have been published about this relationship. Bonovas et al^[34]^ found that current smoking resulted in a significant increase in the risk of POAG (odds ratio [OR] = 1.37, 95% CI: 1.00–1.87), while past smoking history did not appear to affect that risk (OR = 1.03, 95% CI: 0.77–1.38). The analysis included 4 cross-sectional and 3 case–control studies, published during the period leading up to December 2002. The summary OR from a fixed-effects model were 1.37 (95% CI: 1.00–1.87) for current smokers and 1.03 (95% CI: 0.77–1.38) for former smokers. Conversely, other recent meta-analysis did not find this relationship between smoking and glaucoma.^[35]^ Zhou et al^[35]^ found that both current smokers and former smokers were not significantly associated with the risk of POAG. The Zhou meta-analysis included 6 observational studies (3 cohort and 3 case–control studies) from 1 January 1966 to 1 December 2015. The summary relative risk for current smokers was 0.97 (95% CI: 0.81–1.16, *P* = 0.74; I2 = 38%), and for former smokers it was also 0.97 (95% CI: 0.83–1.13, *P* = 0.66; I2 = 46%). These different results indicated that the relationship between smoking and glaucoma is controversial and related to the studies included in each meta-analysis.

The mechanistic action of smoking and whether this could induce glaucoma is not clear and only partially known. Wang et al^[36]^ suggest that smoking alters the biosynthesis of collagen and extracellular matrix turnover, and decreases corneal thickness, which is a risk factor for POAG.^[36]^ Smoking has been reported to decrease the production of oxygen and collagen in tissues during wound healing,^[37,38]^ deteriorating the ocular hypoxia.^[36]^ Zanon-Moreno et al^[39]^ suggest that smoking liberates free radicals that cause damage in the trabecular meshwork, consequently decreasing the outflow of the aqueous humor. Moreover, the mechanism associated with vasoconstriction of the episcleral veins can reduce the aqueous outflow.^[40]^ Another plausible mechanism might be related with inflammation and apoptosis. An experimental study^[39]^ that analyzed aqueous humor and plasma samples of 120 women with POAG found that the levels of interleukin-6 (IL-6), as an inflammation marker, and the expression of caspase-3 and poly ADP-ribose polymerase 1 (PARP-1), as apoptosis markers, were significantly higher in current smokers than in former- and nonsmokers (*P* < 0.05). These data may suggest that smoking could induce the expression of inflammatory and apoptosis markers in patients with glaucoma. Since experimental studies in human^[41]^ and animal models^[42]^ have shown that trabecular meshwork cells and retinal ganglion cells die by apoptosis in glaucoma, it could be argued that the increase of apoptotic and inflammation markers due to smoking in patients with POAG may contribute to the progression of the disease.^[39]^ A biological mechanism might be related to the ischemia caused by tobacco smoking, which is considered a major risk factor in most ischemic disorders of the eye,^[40]^ as it has been suggested by different experimental studies that showed the deleterious effect the tobacco smoking has on ocular blood flow.^[9,43]^ Consequently, since many researchers believe that POAG has a vascular origin based on a compromised blood flow to the optic nerve head,^[44]^ smoking may be involved in the pathogenesis of POAG.

Our study has some important strengths. First, its prospective design implies that information about smoking and other risk factors for glaucoma was obtained before the diagnosis of the disease. Besides, the control of a wide variety of potential confounders was considered prospectively, avoiding the possibility of inverse causation bias, which is a frequent phenomenon in cross-sectional studies. Other advantage is the validation of the diagnosis of glaucoma in a subsample of these participants, as well as the large sample size and the follow-up period (a median of 8.5 years). In addition, both risk factors, namely exposure and outcome, were ascertained through validated questionnaires. Regarding the validity of outcome ascertainment, abundant evidence indicates that self-reported information is valid in our cohort.^[20,45–47]^ However, there are some limitations in our study. Although some degree of misclassification is likely to exist, it is expected to be nondifferential and therefore, to drive the association toward the null value. Our participants did not undergo a systematic ophthalmologic examination to discard the occurrence of glaucoma during follow-up. Some new-onset cases may have been missed leading to a reduced sensitivity. However, theoretically, with perfect specificity, nondifferential sensitivity of disease misclassification will not bias the relative risk estimate.^[48]^ On the other hand, the fact that the cohort recruits mostly university graduates could induce a selection bias. Nonetheless, 50% of our group is composed of health professionals who have access to relevant and accurate information about glaucoma characteristics. Also, this decision is convenient and methodologically adequate in order to better control several other potential biases related to confounding by economic or socio-demographic factors. The method that we applied (ie, restriction) is an excellent technique in epidemiology to prevent these potential sources of confounding since we avoided economic or socio-demographic heterogeneity. Also, we did not separately identify the passive smokers and hence they were included in the nonsmokers group, rendering it impossible to analyze the group of passive smokers in this cohort.

Another specific limitation is that we could not determine the type of glaucoma. However, in the validated subsample of participants all glaucomatous cases were open-angle glaucoma. Also, other outcomes that have been previously validated within the SUN Project have shown good validity.^[20,46,49]^ Another limitation was that the majority of the participants in our sample are Caucasian. This implies that our results may have limits in their extrapolation to populations with higher percentages of minorities, particularly those of African or Caribbean heritage, who are at greater risk of glaucoma.^[7,50]^

In conclusion, in this prospective study we found that current smoking status is associated with a higher risk of developing glaucoma, even though the same association for former smokers or for passive smokers is not clear. In addition, we found a dose–response relationship that demonstrates that the risk of glaucoma increases as the number of cigarette pack-years increases. Nevertheless, further cohorts studies with larger samples of older participants are needed in order to confirm this association.
